# Granulomatous slack skin T-cell lymphoma: an important differential diagnosis with giant cell tumor of soft tissue*

**DOI:** 10.1590/abd1806-4841.20153807

**Published:** 2015

**Authors:** André Ricardo Adriano, Tiago Silveira Lima, Maxime Battistella, Martine Bagot

**Affiliations:** 1 Instituto de Dermatologia Professor Rubem David Azulay - Santa Casa da Misericórdia do Rio de Janeiro - Rio de Janeiro (RJ), Brazil; 2 Hospital Saint Louis, Paris - France

**Keywords:** Lymphoma, Lymphoma, T-Cell, cutaneous, Mycosis fungoides, Soft tissue neoplasms, Sarcoma

## Abstract

Granulomatous slack skin is an indolent T-cell lymphoma, considered to be a variant of mycosis fungoides. Clinically it is characterized by areas of redundant skin, wrinkled, inelastic, with variable erythema and infiltration besides a poikilodermic surface. A differential diagnosis unknown to most dermatologists is the giant cell tumor of soft tissue, which is an extremely rare low-grade sarcoma. The authors report a patient who had undergone extensive surgery because of a primary diagnosis of giant cell tumor of soft tissue, but which proved to be granulomatous slack skin after a second interventional procedure with confirmatory histopathology.

## INTRODUCTION

Lymphomas are predominantly lymph nodes and lymphatic system neoplasms. When these occur on other tissues they are called extranodal lymphomas. In case their onset in on the skin, without evidence of disease in other organs, they are called primary cutaneous lymphomas, with a wide variety of presentations. These can be divided in B and T-cell lymphomas (65% of primary cutaneous lymphomas). They can be also classified by clinical behavior as indolent or aggressive. A detailed classification of primary cutaneous lymphomas was created by the World Health Organization and the European Organization for Research and Treatment of Cancer - WHO-EORTC. The diagnosis is done through clinical and histopathological evaluation and immunohistochemical study.^1-2^

Granulomatous slack skin (GSS) is classified according to WHO-EORTC as a T-cell lymphoma of indolent behavior.^3^ However, the question whether it would represent only a reaction of the host to another hematological neoplasm or even to a benign agent has been raised.^4^ One third of the patients may progress to Hodgkin's disease in the future.^5^ Some studies revealed lesion clonality, a fact that corroborates the indolent cutaneous T-cell lymphoma possibility.

The giant cell tumor of soft tissue (GCT-ST) is an extremely rare low-grade sarcoma, less frequent than the conventional osseous GCT.

We report a clinical case of a patient with a subcutaneous tumor suggestive of GCT-ST, which was revealed to be a GSS.

## CASE REPORT

**Figure 1:**
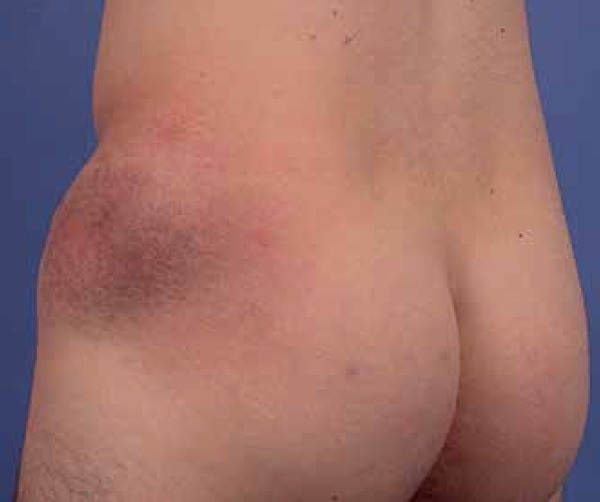
Granulomatous slack skin. Clinical aspect of cutaneous lesion. Erythemato-violaceous, infiltrated and xerotic plaque in the lateral left hip region

Male patient, 25 years old, complaining about the onset of an erythematous lesion in the left hip region when he was 13, having progressed with slow increase in volume since then. At the examination, an erythemato-violaceous, infiltrated and xerotic plaque was observed in the described region (Figure 1). After medical consultation with a general surgeon, a cutaneous biopsy was performed. The histopathological examination showed, in the dermal-hypodermic region, a large inflammatory infiltrate composed mainly of layers of rounded mononuclear histiocytic cells with eosinophilic cytoplasm associated with many multinucleated cells with signs of emperipolesis, besides lymphocitary elements without atypia. He was diagnosed with GCT-ST, with compromised surgical margins.

Magnetic resonance of skin and hip performed in the post-operative period revealed a 20-centimeter area of subcutaneous tissue infiltration, without involvement of muscles or bones. A new approach was necessary, with ample excision, followed by tissue grafting.

**Figure 2:**
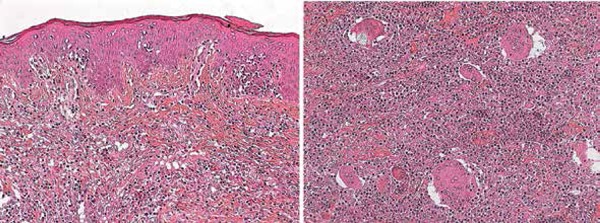
Histopathology of lesion. Extensive lymphoid infiltrate in dermis with discreet epidermotropism, in addition to numerous large multinucleated giant cells (H&E, x100)

**Figure 3:**
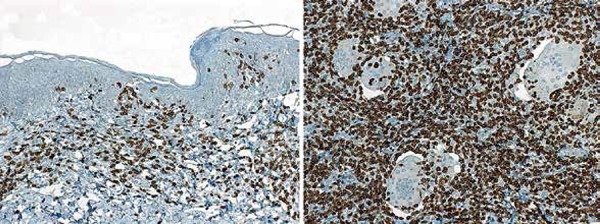
Immuno-histochemistry of lesion. Diffuse positivity with CD4 in the lymphoid cell infiltrate (immunoperoxidase, x100)

Histopathological diagnosis of GSS was then carried out in the new specimen, based on the presence of features already described, associated with extensive, predominantly deep infiltration of lymphocytes, and a CD4 + epidermotropic lymphoid infiltrate on the surface (Figures 2 and 3). Orcein staining revealed the absence of subcutaneous elastic fibers, as well as in deep dermis. Research for T-cell clonality was positive.

## DISCUSSION

GSS is a rare entity with good prognosis, recognized as a variant of mycosis fungoides. It is histologically characterized by the presence of diffuse T-cell infiltrate with numerous multinucleated giant cells, loss of elastic tissue, elastophagia and emperipolesis phenomena. There is also epidermotropism with an infiltrate of atypical lymphocytes.^6^ One can observe, clinically, the progressive development of areas of redundant, wrinkled, inelastic skin, with variable erythema and infiltration, poikilodermic surface and typically plissé, similar to GSS, arising from elastolysis.^6^ Pruritus may be present and there may be ulceration areas. Usually lesions appear on armpits, flanks and groin, sparing hands and eyelids. Incidence is more common in men in their thirties or forties, with indolent progression. Extracutaneous infiltration is seldom found, mostly appearing in the spleen and lymph nodes.^1,4^ Immunophenotyping reveals positivity of CD4 and CD45RO; loss of CD3, CD5 and CD7 markers was also reported.^7^ Giant cells express CD14 and CD68.^4^

Some authors propose that the study and molecular analysis may play an important role in th elucidation of etiopathogeny, diagnosis and prognosis of the disease. They believe that the rearranging of T-beta cell receptor gen is the first step in neoplastic transformation of GSS, and that trisomy of chromosome 8 found in tumor cells would indicate malignant progression. This would favor the hypothesis of GSS malignancy.^6^ However, other authors reported a case of GSS with no evidence of T-cell clonality and rearrangement of T-beta cell receptors, suggesting that not all cases are indolent lymphomas, but a spectrum of diseases that may progress to a lymphoproliferative process.^7^

GSS is an indolent entity in already described cases with more than 10 years of progression. It is not life-threatening, however its association with other neoplasm changes the prognosis.^4^

A differential diagnosis that deserves attention is Granulomatous Mycosis Fungoides (GMF). In this entity nodules and plaque can be observed all over the skin, without preference for flexural areas.^4^ Cutaneous manifestations are similar to classical mycosis fungoides.^8^ Moreover, GMF may spare subcutaneous tissue, different from GSS. In GSS there are numerous multinucleated giant cells, many of them with more than 40 nuclei per cell, throughout the lesion; in GMF the presence of these cells is focal and the number of nuclei is much greater, between 5 and 10. Pautrier's microabscesses are present in GMF, whereas lymphophagocytosis and reduction of elastic fibers is characteristic of GSS.^4^ GMF displays poor response to topical therapeutics, requiring early systemic treatment.^8^

Choosing the treatment for GSS will depend on the stage of disease. There is no standard protocol. Topical nitrogen mustard, PUVA, radiotherapy, corticosteroids, retinoids, interferon, chemotherapy, surgical excision or a combination of these may be used.^9^

There are authors that point to positron emission tomography as an excellent tool for evaluating disease progression, involvement of other tissues and response to treatment.^9^

On the other hand, the giant cell tumor of soft tissue (GCT-ST) is characterized by nodules of mononuclear histiocytes in a vascular stroma, with osteoclast-similar multinucleated giant cells. This pattern of histiocytic lesion was what caused the diagnostic confusion.^10^

Conventional differential diagnoses of GCT-ST are atypical fibroxanthoma, an extension for soft tissues of osseous giant cell tumor, extraosseous osteosarcoma, osteoclastic giant cell leiomyosarcoma and malignant fibrous histiocytoma.

GSS is a little known differential diagnosis of GCT-ST and cases are mainly referred to surgeons. We highlight the necessity of habitual referring of cutaneous lesions to the dermatologist, who by including the clinical hypothesis of GSS would facilitate a diagnosis by the pathologist. Clinical recognition of this entity would avoid the patient undergoing unnecessary surgeries.
